# Ultra‐Low‐Dose Isotretinoin for Acne: A Pilot Evaluation of Efficacy and Tolerability in Chinese Patients

**DOI:** 10.1111/jocd.70805

**Published:** 2026-03-24

**Authors:** Suhong Shi, Jinfeng Liu, Lingyu Pan, Wei Hua

**Affiliations:** ^1^ Department of Dermatovenereology West China Hospital, Sichuan University Chengdu China; ^2^ Cosmetics Safety and Efficacy Evaluation Center West China Hospital, Sichuan University Chengdu Sichuan China

To the Editor,

Acne vulgaris substantially compromises quality of life across all severity levels [1, 2]. Although conventional isotretinoin dosing (0.5–1 mg/kg/day) demonstrates high efficacy, this regimen may represent overtreatment for milder cases and is associated with significant adverse effects and economic burden [3]. Consequently, modified dosing strategies (intermittent 0.5 mg/kg/week or fixed 10–20 mg/day) have been implemented to enhance tolerability and cost‐efficiency [4, 5]. Our preliminary clinical observations suggested potential therapeutic benefits with doses below 0.1 mg/kg/day; however, robust evidence regarding their efficacy and safety profile remains scarce.

To investigate this further, we conducted a pilot study examining ultra‐low‐dose isotretinoin (10 mg administered either every other day or twice weekly) in a real‐world cohort of 44 acne vulgaris patients over 12 weeks. Physicians could adjust dosing based on tolerability or laboratory parameters, while concomitant use of topical therapies (clindamycin gel, benzoyl peroxide, and azelaic acid) was permitted. Clinical evaluations were performed at baseline, week 4, and week 12, with acne severity assessed using the Global Acne Grading System (GAGS) and patient satisfaction measured via a Subject Satisfaction Score (SSS) [6]. Safety monitoring included liver function tests and lipid profiles at week 4.

Among the 44 enrolled patients, 42 (mean age 22.21 ± 3.33 years, 36 female) completed the 12‐week follow‐up and were included in the final analysis, while two were lost to follow‐up. Baseline characteristics and the calculated weight‐based daily doses are summarized in Table 1. The median daily dose across all patients was 0.086 (IQR 0.071–0.092) mg/kg/day, with all doses falling below 0.104 mg/kg/day, confirming the ultra‐low‐dose nature of the intervention.

**TABLE 1:** Baseline characteristics and ultra‐low‐dose isotretinoin regimens.

Variable	Value (*N* = 42)
**Demographics**	
Female, *n* (%)	36 (85.71%)
Age, years, mean ± SD	22.21 ± 3.33
Body weight, kg, mean ± SD	55.48 ± 5.29
**Disease characteristics**	
Disease duration, years, median (IQR)	5.0 (3.0–8.25)
Baseline GAGS^a^ score, median (IQR)	22.00 (17.50–27.00)
**Acne severity by GAGS at baseline, *n* (%)**	
Mild (1–18)	10 (23.81%)
Moderate (19–30)	25 (59.52%)
Severe (31–38)	6 (14.29%)
Very Severe (≥ 39)	1 (2.38%)
**Treatment regimens & dosage**	
10 mg every other day	31 (73.81%)
10 mg twice weekly	7 (16.67%)
10 mg every other day, reduced to twice weekly	4 (9.52%)
**Calculated daily dose** ^**b**^, **mg/kg/day, median (IQR)**	0.086 (0.071–0.092)

^a^ GAGS, Global Acne Grading System.

^b^ Daily dose calculated as: (dose per intake × weekly frequency)/(7 × individual body weight).

A marked reduction in acne severity was observed (Table 2), with median GAGS scores declining from 22.00 (IQR 17.50–27.00) at baseline to 17.50 (IQR 12.25–21.25) at week 4 (*p* < 0.001) and further to 7.00 (IQR 5.00–10.00) at week 12 (*p* < 0.001). Patient satisfaction was notably high, with 66.7% (28/42) reporting “satisfied” or “very satisfied” at week 4, increasing to 97.62% (41/42) by week 12.

**TABLE 2:** Efficacy and safety outcomes.

Outcome measure	Value (*N* = 42)
**Efficacy**	
**GAGS** ^**a**^ **score, median (IQR)**	
Baseline	22.00 (17.50–27.00)
Week 4	17.50 (12.25–21.25)^b^
Week 12	7.00 (5.00–10.00)^b^
**Subject satisfaction score (SSS)** ^**c**^ **at Week 4, *n* (%)**	
Very satisfied	9 (21.43%)
Satisfied	19 (45.24%)
No difference	14 (33.33%)
Unsatisfied/very unsatisfied	0 (0%)
**Subject satisfaction score (SSS) at Week 12, *n* (%)**	
Very satisfied	18 (42.86%)
Satisfied	23 (54.76%)
No difference	1 (2.38%)
Unsatisfied/very unsatisfied	0 (0%)
**Safety & Tolerability**	
Most common adverse events, *n* (%)	
Cheilitis	13 (31.0%)
Dry skin	10 (23.8%)
Dry eyes	1 (2.4%)
Other^d^	4 (9.5%)
Laboratory abnormalities (Week 4), *n* (%)	
Mild ALT/AST elevation	3 (7.14%)
Hypertriglyceridemia	0 (0%)

^a^ GAGS, Global Acne Grading System.

^b^ *p* < 0.001, Wilcoxon signed‐rank test, compared with Baseline.

^c^ Subject Satisfaction Score, consists of five levels: +2, very satisfied; +1, satisfied; 0, no difference; −1, unsatisfied; −2, very unsatisfied.

^d^ Includes skin pruritus, skin tingling, fatigue, and alopecia (each *n* = 1).

The treatment regimen exhibited a favorable safety and tolerability profile. Mild, asymptomatic elevations in alanine aminotransferase or aspartate aminotransferase were observed in three patients (7.14%) during the week 4 laboratory assessment. Following dose reduction, all biochemical abnormalities resolved by week 12. No instances of hypertriglyceridemia were detected, and no treatment discontinuations occurred due to laboratory abnormalities. The most common adverse events were mucocutaneous in nature, including dry skin (*n* = 10), cheilitis (*n* = 13), and dry eyes (*n* = 1). Other minor adverse events comprised skin pruritus (*n* = 1), skin tingling (*n* = 1), fatigue (*n* = 1), and alopecia (*n* = 1). All reported events were mild in severity and clinically manageable.

In this pilot real‐world study, we evaluated the feasibility, tolerability, and preliminary outcomes of ultra–low‐dose isotretinoin administered at low‐frequency dosing schedules in patients with acne vulgaris. The aim was not to establish causal efficacy, but to assess whether doses substantially lower than those commonly reported could provide acceptable clinical improvement with reduced treatment burden. Our regimen represents an ultra–low‐dose approach rarely characterized in prior studies. Concomitant topical therapies, reflecting routine practice, limit efficacy interpretation; thus, findings should be viewed as the incremental value of isotretinoin added to standard care. Safety outcomes were favorable, with mild and reversible adverse events. Despite limitations, this approach appears feasible and well‐tolerated, warranting further controlled studies.

## Consent

This study was conducted in accordance with the principles of the Declaration of Helsinki. The study protocol was reviewed and approved by the Ethics Committee of West China Hospital, Sichuan University (Approval No. 2025‐1340). Written informed consent was obtained from all participants prior to their enrolment in the study.

## Conflicts of Interest

The authors declare no conflicts of interest.

## Data Availability

The data that support the findings of this study are available on request from the corresponding author. The data are not publicly available due to privacy or ethical restrictions.
